# The Influence of Co Additive on the Sintering, Mechanical Properties, Cytocompatibility, and Digital Light Processing Based Stereolithography of 3Y-TZP-5Al_2_O_3_ Ceramics

**DOI:** 10.3390/ma13122789

**Published:** 2020-06-20

**Authors:** Margarita Goldberg, Tatiana Obolkina, Sergey Smirnov, Pavel Protsenko, Dmitriy Titov, Olga Antonova, Anatoliy Konovalov, Egor Kudryavtsev, Irina Sviridova, Valentina Kirsanova, Natalia Sergeeva, Vladimir Komlev, Sergey Barinov

**Affiliations:** 1 Laboratory of Composite Ceramic Materials, A.A. Baikov Institute of Metallurgy and Materials Science, Russian Academy of Sciences, 119334 Moscow, Russia; tobolkina@imet.ac.ru (T.O.); ssmirnov@imet.ac.ru (S.S.); protsenko@colloid.chem.msu.ru (P.P.); dtitov@imet.ac.ru (D.T.); oantonova@imet.ac.ru (O.A.); akonovalov@imet.ac.ru (A.K.); komlev@mail.ru (V.K.); barinov_s@mail.ru (S.B.); 2 Department of Chemistry, M.V. Lomonosov Moscow State University, 119991 Moscow, Russia; 3 Joint Research Center, Technology and Materials, Belgorod State National Research University, 308015 Belgorod, Russia; egoryoda@mail.ru; 4 Laboratory of Prediction of Cancer Treatment Response, National Medical Research Radiological Center of the Ministry of Health of the Russian Federation, 125284 Moscow, Russia; i.k.sviridova@yandex.ru (I.S.); kirik-57@mail.ru (V.K.); prognoz.06@mail.ru (N.S.)

**Keywords:** ZrO_2_, Al_2_O_3_, Co oxides, CoAl_2_O_4_, sintering additives, DLP, in vitro, mechanical properties

## Abstract

Nanocrystalline 3 mol% yttria-tetragonal zirconia polycrystal (3Y-TZP) ceramic powder containing 5 wt.% Al_2_O_3_ with 64 m^2^/g specific area was synthesized through precipitation method. Different amounts of Co (0–3 mol%) were introduced into synthesized powders, and ceramic materials were obtained by heat treatment in the air for 2 h at 1350–1550 °C. The influence of Co addition on the sintering temperature, phase composition, microstructure, mechanical and biomedical properties of the obtained composite materials, and on the resolution of the digital light processing (DLP) printed and sintered ceramic samples was investigated. The addition of a low amount of Co (0.33 mol%) allows us to decrease the sintering temperature, to improve the mechanical properties of ceramics, to preserve the nanoscale size of grains at 1350–1400 °C. The further increase of Co concentration resulted in the formation of both substitutional and interstitial sites in solid solution and appearance of CoAl_2_O_4_ confirmed by UV-visible spectroscopy, which stimulates grain growth. Due to the prevention of enlarging grains and to the formation of the dense microstructure in ceramic based on the tetragonal ZrO_2_ and Al_2_O_3_ with 0.33 mol% Co the bending strength of 720 ± 33 MPa was obtained after sintering at 1400 °C. The obtained materials demonstrated the absence of cytotoxicity and good cytocompatibility. The formation of blue CoAl_2_O_4_ allows us to improve the resolution of DLP based stereolithographic printed green bodies and sintered samples of the ceramics based on ZrO_2_-Al_2_O_3_. The developed materials and technology could be the basis for 3D manufacturing of bioceramic implants for medicine.

## 1. Introduction

Yttria-tetragonal zirconia polycrystal (Y-TZP) is widely used as construction ceramic material for automotive, aerospace, and other engineering applications due to its hardness, high strength, durability, and chemical resistance [1]. Additionally, Y-TZP is currently applied as material for stomatology, implants for hip joints, and as a coating material for endoprostheses [2]. The introduction of Al_2_O_3_ in Y-TZP resulted in a decrease in the recrystallization rate, keeping the particle size of the tetragonal-ZrO_2_ (t-ZrO_2_) within the critical size without transformation into monoclinic modification [3]. Introduction of up to 5 wt.% Al_2_O_3_ resulted in a decrease in low temperature degradation of 3Y-TZP, which is dramatically detrimental for the biomedical application of Y-TZP ceramics [4,5]. Composite materials based on Y-TZP and Al_2_O_3_ are characterized by enhanced mechanical properties due to Al_2_O_3_ high elastic modulus and a decrease of composite materials grain size, but, at the same time, the sintering of these materials occurs at a higher temperature (1600 °C for 3Y-TZP-20 wt.% Al_2_O_3_ [6], 1650 °C for 3Y-TZP- 35, 40, 45, 50, 55, 60 and 65 wt.% Al_2_O_3_ [7], 1500–1600 °C for 3Y-TZP-90 vol% Al_2_O_3_ [8]) comparing to 1450 °C for pure Y-TZP according to Li et al. [9]. This disadvantage could be compensated in different ways, for example, using hot isostatic pressing, spark plasma sintering, microwave sintering, longer sintering (up to 96 h), or by sintering additives introduction [6,10,11,12,13]. The influence of 1.0, 3.0 and 5.0 mol% of the different cations—Co, Ni, Fe, Li, Bi, Zn, Mn, Ga, Gd—on the sintering of cubic ZrO_2_ was studied in [14]. The influence on the intensification of the sintering kinetics was observed for Bi, Li, Fe, and in a less degree for Co, Mn, Ni, and Zn without destabilizing the cubic 8Y-TZP crystal structure. It was linked to the formation of crystal lattice defects with the introduction of the cation with different ionic radius and valency, comparing to Zr^4+^. At the same time, the data on the metal oxides’ influence on the sintering of tetragonal modification of ZrO_2_ (t-ZrO_2_) is limited up to date. The introduction of Fe oxide demonstrated the improvement of the densification and mechanical properties of the Y-TZP with 10 and 20 wt.% of Al_2_O_3_ at 1450–1500 °C [15].

Currently, digital light processing (DLP) based stereolithography 3D manufacturing is considered as a powerful technique to elaborate ceramic materials with complex geometrical shape [16,17,18,19]. The interaction between photopolymer and light is dependent on the color of the slurry [20]. For the improvement of the printing resolution, the color additives were investigated, and the creation of the blue tint by the introduction of CoAl_2_O_4_ into commercial 3 mol% Y_2_O (3Y-TZP) slurry resulted in a decrease of cure depth and an increase of relative density of ceramic materials [9]. Cobalt is one of the promising cations for defects formation in the ZrO_2_ due to a significant difference in ionic radius and multivalence of Co oxides, moreover, Co based compounds are characterized by primarily blue color [20]. Hartmanová et al. [21] reported the improvement in the densification of 12 mol% yttria-stabilized cubic zirconia (12-YSZ) when up to 0.5 wt.% of Co_2_O_3_ was introduced, but resulted materials still had high porosity over 10%. The authors observed a maximum of electrical conductivity at 0.1 mass% Co_2_O_3_. Lewis et al. [22] demonstrated improved densification in 8-YSZ with Co_3_O_4_ addition. However, these papers did not discuss the influence of Co addition on the mechanical properties of YSZ ceramic materials, because its potential application in the high-temperature solid oxide fuel cells does not require high strength. At the same time, mechanical properties are essential for engineering and biomedical application. To our knowledge, there is no data on Co concentration influence on the sintering of Y-TZP-Al_2_O_3_ composite materials. The influence of Co additive on the resolution of the DLP printed ceramic samples will be also the object of the investigation. Finally, the impact of Co doping of Y-TZP on the biocompatibility of resulted ceramics is important for potential biomedical applications. Data on the subject was recently reviewed in [23,24].

The starting ceramics composition of 3Y-TZP-6.1 mol% Al_2_O_3_ was selected according to the following: (i) 3 mol% of Y_2_O_3_ is sufficient and essential to stabilize tetragonal phase, increase of yttria content can provoke cubic phase formation [25]; (ii) addition of 6.1 mol% Al_2_O_3_ (5 wt.%) is sufficient to improve mechanical properties of ceramic composition [14]. To summarize, this paper aimed to establish the influence of Co addition on the sintering temperature, phase composition, microstructure, and mechanical properties of the composite materials based on 3Y-TZP-Al_2_O_3_. The first data on the DLP printing and sintering of these ceramic materials will be presented, and the influence of blue coloration of Co-doped ceramic (confirmed by UV-visible reflectance spectra) on their properties will be discussed.

## 2. Materials and Methods

### 2.1. Powders Synthesis and Ceramic Sintering

Powders of the 3Y-TZP-5Al2O3 were obtained by the chemical co-precipitation method. All salts were analytically pure, produced by Labtech. The mixture of ZrOCl_2_·8H_2_O (248.3 g), YCl_3_·6H_2_O (14.5 g) (corresponding to a composition of 97 mol% ZrO_2_ and 3 mol% Y_2_O_3_) and AlCl_3_·6H_2_O (24.04 g) dissolved in 450 mL of distilled H_2_O was added with stirring to the solution of NH_4_HCO_3_ (33.3 g) in the mixture of 200 mL of 25% NH_4_OH and 170 mL of H_2_O at room temperature. The pH level during the synthesis was 8–9. The obtained reaction mixture was stirred for 5 min, dried, and calcined at 650 °C in air. After calcination, the powders were ground in a planetary ball mill for 30 min in the ethanol media, washed with ethanol on Buchner funnel and air-dried. At this stage, cobalt oxide was added. For this purpose, the obtained powders were introduced in the CoCl_2_ aqueous solution, mixed for 1 h, evaporated, air dried, and bolted through a capron sieve with mesh size of ~100 × 100 μm. The concentrations of Co in the solutions were taken to achieve the target amount of metal in mol% in the ceramic materials. There were four concentration of cobalt: 0 mol% Co (composition I), 0.33 mol% Co (composition II), 1.0 mol% Co (composition III) and 3.0 mol% Co (composition IV). Stoichiometry of the samples was achieved through the ratio of initial components. The powders were pressed in a metallic mold at 100 MPa using uniaxial compression and sampled in the form of bars 30 × 4 × 4 mm and cylinders 0.5 × 8 mm were obtained. The bars were densified by pressureless sintering in the air at a temperature range of 1350—1550 °C for 2 h. Porosity, density, phase composition, mechanical properties and microstructure of the bar samples were investigated. Before mechanical tests and microscopy investigations, all specimens were polished using a set of diamond paste down to 0.5 µm. The cylinder samples were applied for dilatometry study.

### 2.2. Ceramic Samples DLP Printing and Sintering

Samples were printed using WANHAO (Precision Casting Co., Ltd. 77, Renming Road, Jinhua, China) with λ = 405 µm. The powders were sieved through nylon sieve with 50 µm mesh to avoid the agglomeration. Oligoester acrylate photopolymer was mixed with obtained powder via the ultrasound mixing for 25 s with a photopolymer: powder mass ratio of 1:1. The printing depth was 35 µm per layer after the exposition of 180 s. After the printing, the green samples were ultrasound treated in the ethanol media for removing non-reacted photopolymer. The green samples were debinded for photopolymer removal by thermal decomposition in the airflow by gradual heating up to 1000 °C during 20 h. This procedure was slightly modified variant of the program presented in [26]. The samples were sintered for 2 h at 1450 °C. The microstructure and the linear shrinkage were established.

### 2.3. Characterization Techniques

The particle morphology of 3Y-TZP powders before Co introduction was assessed by TEM (JEM 2100, JEOL, Japan, carbon sputtered specimens). The specific surface area (S) of the as-synthesized powders was determined by low-temperature nitrogen adsorption measurements (BET, TriStar analyzer, Micromeritics, GA, USA). The powder materials were characterized by the X-ray diffraction (XRD) method (Shimadzu XRD-6000, CuKα radiation λ = 1.54184 Å, step = 0.02°) with the identification of phase composition according to JCPDS database. Cell parameters of t-ZrO_2_ phase were estimated by means of FullProfSuite software(Version 7.30, France). Dilatometric investigations were carried out at temperatures up to 1500 °C with 10 °C/min rate in Ar atmosphere 70 mL/min flow using dilatometer with vacuum-tight oven and working up to 1650 °C (Dil 402 C dilatometer, Netzsch, Germany). The investigated sample was placed into a horizontal corundum holder equipped with a corundum pusher with a high-precision displacement transducer (measuring range 500–5000 μm). Corundum spacers were placed between the sample and pusher. In all cases, vacuum-tight corundum (Al_2_O_3_—98.7%, TiO_2_—1%, SiO_2_—0.3%), which was characterized by high chemical resistance, was used.

The diffuse reflection coefficients (R) and L × a × b × color parameters were recorded using a UV-vis minispectrometer (Eye-One Pro 2, X-Rite, USA). The L × a × b × color parameters of the powders and ceramics samples were measured using the standard lighting C, following the CIE-L × a × b × colorimetric method recommended by the Commission Internationale de l’Eclairage (CIE) [27]. The diffuse reflection coefficients were measured in a range of 400–730 nm with the 10 nm increment. Then, the values of the Kubelka–Munk function (F) were calculated according to the equation:

F = (1 − R^2^)/2R (1)

where R is the diffuse reflection coefficient. The plotted spectra F versus λ were used to reveal characteristic adsorption of Co-based substances. b* chromaticity coordinate was used to estimate the blueness of the samples. The density of sintered samples was measured by hydrostatic weighing with ABS weighing-machine. The accuracy of determination was better than 0.05%. The relative density was calculated with respect to the theoretical density of tetragonal ZrO_2_ (6.08 g/cm^3^) [28], and α-Al_2_O_3_ (3.99 g/cm^3^) [29]. The bending strength was measured on the polished bars, the dimensions of the specimens were 25 × 3 × 2.5 mm after polishing, 5 samples for each point (Instron 3382 pull-test machine, Norwood, MA, USA). A 3-point fixture was used, where the span distance was 10 mm and the cross-head speed was 1.0 mm/min. The microhardness was measured by the Vickers method (401/402-MVD Instron Wolpert Wilson Instruments) with the load of 1960 mN for 10 s. The microstructure of the ceramic materials was studied using scanning electron microscopy (SEM, Tescan Vega II, Czech Republic). For this purpose, the polished ceramic samples were thermally etched at Tetc = Tsintering − 100 °C [30] and coated with a thin layer of gold to prevent surface charging (Q150R ES, Quorum Technologies, East Sussex, UK). The ceramic materials were investigated with TEM (JEM 2100, JEOL, Japan). For this purpose, the ceramic samples were grounded in an agate mortar, the particles were sieved through nylon sieve with 30 µm mesh and investigated as powders with preparing of the suspensions in isopropyl alcohol, which were applied on the copper mesh coated with a carbon film according to the scheme described in [31]. The phase composition was confirmed by the electron diffraction (ED) data obtained by TEM.

### 2.4. Cytotoxicity Studies

*In vitro* cytotoxicity of the sintered at 1450 °C samples was investigated on the cell line of human osteosarcoma MG-63 (Russian Collection of Cell Cultures, Institute of Cytology, Russian Academy of Sciences, St. Petersburg, Russian Federation). The cells were tested for the absence of mycoplasma using the PCR method. A day before the experiment, a suspension of MG-63 (seeding density 3.5 × 10^4^ cells per ml) was placed into a 96-well plate (Corning Costar, Merch, USA) in triplets per sample in 200 μL of cells growth media (CGM) of the following composition: DMEM medium (PanEco, Moscow, Russia), 10% fetal bovine serum (PAA, Austria), 60 mg/mL-glutamine (PanEco, Moscow, Russia), 20 mM Hepes buffer (PanEco, Moscow, Russia) and 50 μg/mL gentamycin (PanEco, Moscow, Russia). Cell incubation was carried out at 5% CO_2_ and 37 °C (CO_2_ incubator, Sanyo, Japan).

The cytotoxicity of the sterile samples (treated at 180 °C during 1.5 h in Binder, USA) was determined in accordance with ISO 10993.5-99 by direct contact of the extract (0.2 g of material in 1 mL of CGM) with the test culture. Extraction was carried out for 24 h at a temperature of 37 °C with constant stirring on an orbital shaker (Elmi, Latvia). The pH values of the solution were determined for each sample of extracts (Hanna, Germany). To assess the possible toxicity of the extracts of the obtained ceramic samples, after 24 h of incubation MG-63 growth medium was taken from the wells with the test culture and 200 μL of the obtained extracts were replaced. As a control, pure CGM was used.

The viability of the MG-63 culture was determined after 24, 48, and 72 h using the MTT test [32]. The MTT test is based on the ability of dehydrogenase of living cells to reduce 3-(4,5-dimethyl-2-thiazolyl)-2,5-diphenyl-2h-tetrazolium bromide (MTT, (Sigma), USA) into formazan. The optical density of formazan, which is produced by the mitochondrial activity of viable cells from MTT, was measured at 540 nm using a Multiskan FC microplate photometer (Thermoscientific, USA).

For each sample extracts, the toxicity index (TI) was calculated according to ISO 10993.5-99 by the Equation (2):

TI = 100% − OD exp/ODcontrol (%)(2)

where OD is the optical density of the formazan solution in the experiment and the control, respectively and their ratio essentially represents a pool of viable cells (PVC). A sample of the material was considered as a non-toxic when an IT value is lower than 30%. The obtained results were processed by conventional methods of variational statistics using Microsoft Excel 2000. The significance of differences was assessed using a parametric Student t-test; differences were considered statistically significant at p < 0.05.

## 3. Results

### 3.1. Powders Morphology, Surface Area, and Phase Composition before Co Introduction

**Figure 1:**
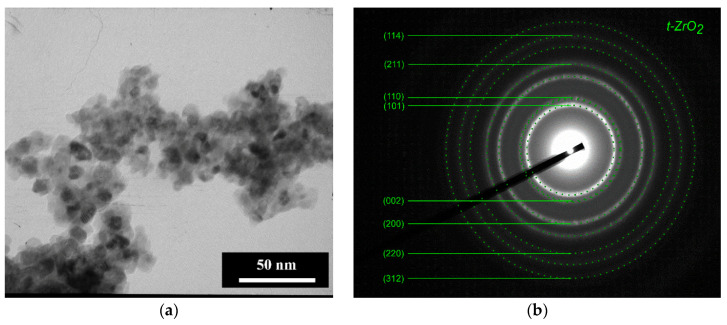
TEM image of pure 3Y-TZP-Al_2_O_3_ powder (composition I) (**a**) and ED with Miller indexes (**b**).

According to TEM data, the particles of 3Y-TZP-5Al_2_O_3_ powders were round-shaped with a 10–20 nm average diameter (Figure 1a). The degree of aggregation was low due to the milling of obtained powders as it was described in [33]. According to BET gas adsorption data, the pure 3Y-TZP-Al2O3 composite had the specific area S of 64 ± 0.5 m^2^/g. Considering the spherical morphology, we have calculated the particle size according to the simple equation

(3) $$d=\frac{6}{S\rho }$$

where d—particle size (nm), S—specific area (m^2^/g), $\rho^{\prime }$—the value of a theoretical density which was assumed to be 5.97 g/cm^3^, based on the theoretical density values of 3Y-TZP (6.08 g/cm^3^) and Al_2_O_3_ (3.99 g/cm^3^). The average particle size was 15 nm, in agreement with the TEM data indicating the dense structure of observed particles.

The Miller indexes are presented in Figure 1b. According to ED, the main phase was tetragonal ZrO_2_ (t-ZrO_2_) with the reflexes assigned to (101) at 2.97 Å; (002) at 2.61; (110) at 2.5 Å, (200) at 1.81 Å (JCPDS card #79-1768). It is hard to separate reflexes (002) and (110), reflex (200) is probably overlapped with (112). Reflexes with lower intensities were attributed to t-ZrO_2_ as well.

**Figure 2:**
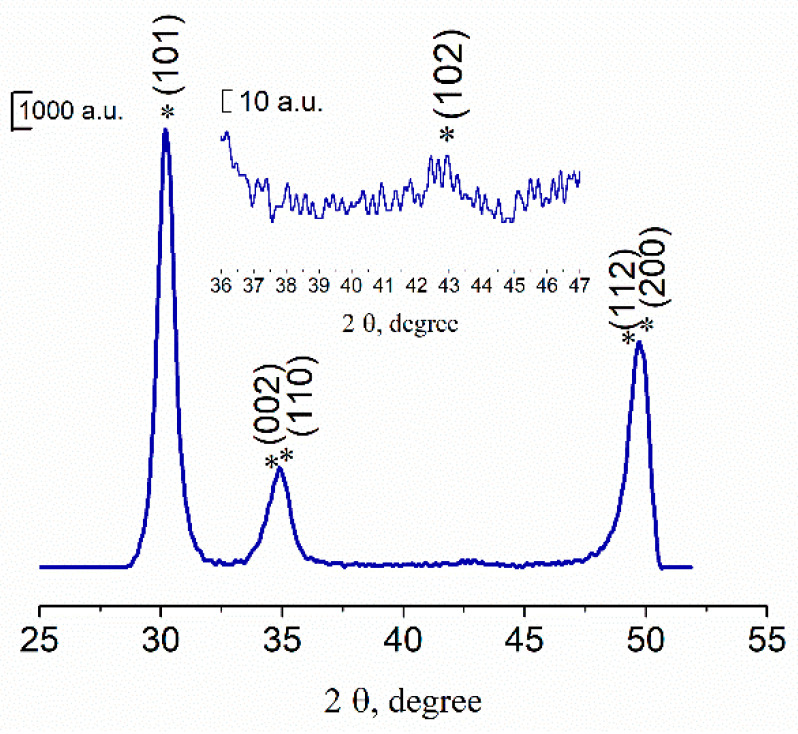
XRD data of pure 3Y-TZP-Al_2_O_3_ powder (composition I), where * is t-ZrO_2_.

The XRD data confirmed the electron diffraction results that powders after thermal treatment at 650 °C were formed by low-crystallinity t-ZrO_2_ (JCPDS card #79-1768) (Figure 2). The broad peak at 2θ ≈ 35° corresponds to overlapped reflexes of (002) and (110) and at 2θ ≈ 50°—to (200) and (112). There were no peaks of Al_2_O_3_ in the diffractogram due to the low crystallinity degree of powders. Previously, it was demonstrated that the only crystalline phase in the Y-TZP-Al_2_O_3_, when Y-TZP content was over 75 mol%, was t-ZrO_2_ [34].

### 3.2. Dilatometric Study of the Ceramic Samples

**Figure 3:**
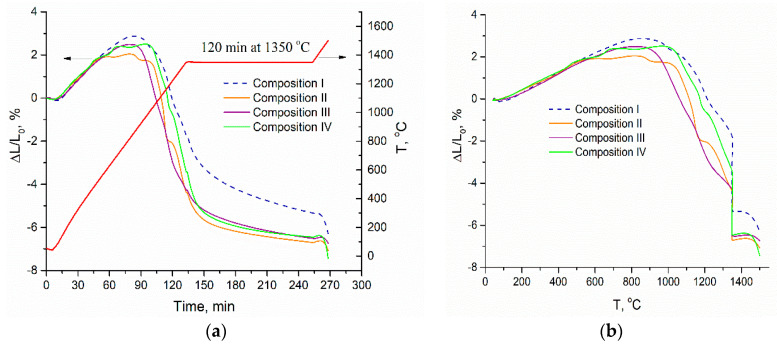
Sintering curves of 3Y-TZP-Al_2_O_3_ for Compositions I–IV with continuous heating and isothermal exposure at 1350 °C. Shrinkage is plotted vs. time together with heating profile (**a**) and vs. temperature (**b**).

We can see the variation of the shrinkage with time for 3Y-TZP-Al_2_O_3_ samples with different Co concentration in Figure 3. The dilatometric analysis data demonstrate that the sintering process has a multistage character. For the T < 600 °C no shrinkage was observed, enlargement of the samples is due to thermal expansion and relaxation of initially compressed samples. The introduction of Co additive resulted in the shift of shrinkage initiation from ~750 to 550–600 °C. Between 1100 and 1350 °C shrinkage rate is compatible for pure 3Y-TZP-Al_2_O_3_ and samples containing Co. As the last start to shrink earlier the total shrinkage for them was 3.4–4.4% against 1.7% for pure 3Y-TZP-Al_2_O_3_. After isothermal holding at 1350 °C for 2 h, all Co doped samples shrink to 6.3–6.7% and pure sample to 5.3%. Further increase of temperature up to 1500 °C lead to additional shrinkage of Co doped samples to 6.7–7.5% and pure sample to 6.3%. So, the addition of Co stimulates shrinkage in a wide temperature range, the influence of Co concentration was not revealed. The fact that we cannot reveal the difference in shrinkage for different Co concentrations could be linked to the effect of CoAl_2_O_4_ formation from the excess of Co.

### 3.3. Phase Composition of the Ceramics Samples

**Figure 4:**
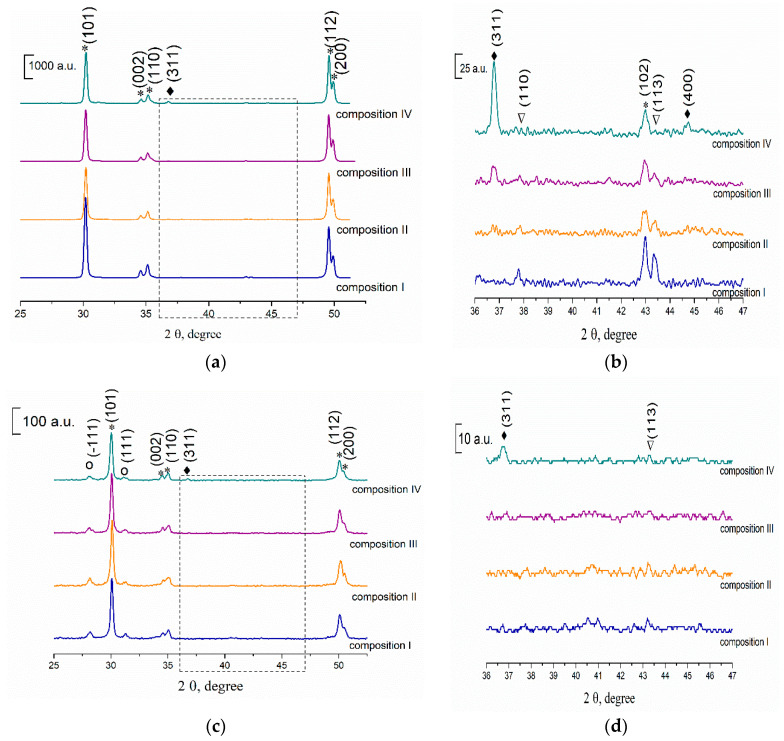
XRD spectra of 3Y-TZP-Al_2_O_3_ ceramics sintered at: (**a**,**b**) 1350; (**c**,**d**) 1450 °C, where *—t-ZrO_2_, o—m-ZrO_2_, ∇—Al_2_O_3_, ♦—CoAl_2_O_4_.

**Figure 5:**
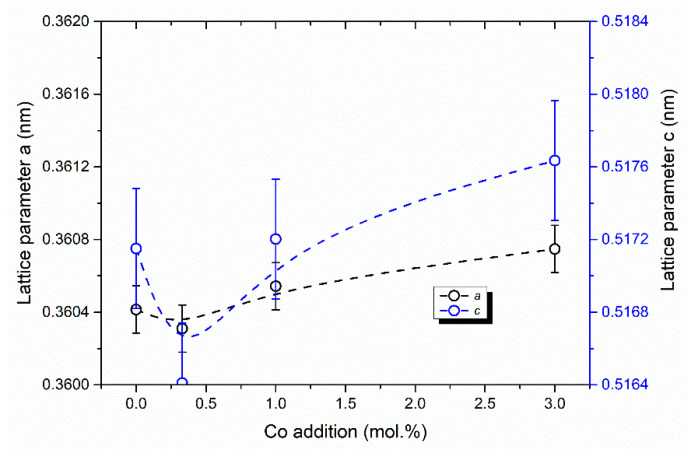
The effect of the Co amount on the cell parameters on the samples sintered at 1350 °C.

According to XRD data, the zirconia in all samples after sintering at 1350 °C was presented by the only t-ZrO_2_ (JCPDS card #79-1768) (Figure 4a,b). The intensity of the main peak of Al_2_O_3_ (JCPDS card #10-0173) at 43.36 2θ with the plane indexes (113) was very weak and it decreased with full disappearing when Co content growth up to 3.0 mol% (composition IV). This was linked with the formation of spinel CoAl_2_O_4_ (JCPDS # 44-0160) which was observed for compositions III and IV as a result of Co ions interaction with Al_2_O_3_ at sintering temperature (Figure 4). This result agreed with the data, reported in [35], where the formation of CoAl_2_O_4_ was detected when 2.4 mol% of CoO and 1.5 wt.% Al_2_O_3_ were introduced in 3Y-TZP powders. In the case of composition II, the spinel was not detected by XRD but could be formed in an amorphous state or on the grain boundaries. In work [36] it was demonstrated that in CoO-Al_2_O_3_ mixture CoAl_2_O_4_ was formed at T > 1050 °C even for as low concentration of Co as 1.0 mol%. Cell parameters of ceramics sintered at 1350 °C for t-ZrO_2_ phase were calculated for compositions I–IV. The results were presented in Figure 5. The volume of the elementar cell is minimum for composition II. This effect is linked with the formation of the solid solution based on the t-ZrO_2_. A similar behavior was observed by Hartmanova et.al. [21] for cubic ZrO_2_ with Co_2_O_3_ additive and it was explained by the consequent filling of the two types of Co sites in the ZrO_2_ lattice. The formation of the substitutional type of ZrO_2_-Y_2_O_3_-Co_2_O_3_ solid solution observed up to 0.2 wt.% of additive, and further doping resulted in both the substitutional and interstitial positions occupation. According to our data, we observed the substitutional solid solution type when 0.33 mol% of Co was introduced, and starting from the 1 mol% of Co the mixed type of solid solution was formed. To our knowledge, the influence of Co introduction on the cell parameters of the t-ZrO_2_ was demonstrated for the first time.

The phase compositions did not change significantly at 1400 °C. The further increase of sintering temperature up to 1450 °C resulted in the formation of the low-strength m-ZrO_2_ (JCPDS #37-1484) (Figure 4d,e). The intensity of t-ZrO_2_ peaks decreased with the growth of Co amount which indicates that transformation of t-ZrO_2_ into m-ZrO_2_ is promoted by Co. This effect was linked with significant grains growth in the presence of CoAl_2_O_4_ resulting in the formation of grains with critical size for the transformation of t-ZrO_2_ into m-ZrO_2_, as it is demonstrated by SEM (see below). At the same time, the amount of Al_2_O_3_ and CoAl_2_O_4_ phases decreases noticeably when temperature increased from 1350 to 1450 °C indicating dissolution of these phases into ZrO_2_. The further increase of sintering temperature up to 1500–1550 °C resulted in continuous growth of the m-ZrO_2_ phase amount for all compositions.

**Figure 6:**
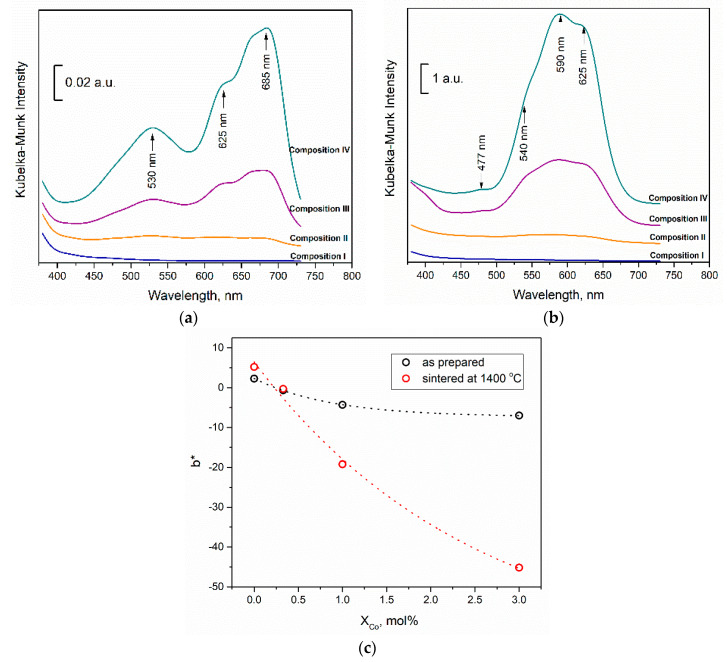
UV–vis absorption spectra of the powders (**a**) and sintered at 1400 °C ceramics (**b**) dependent the composition, plot of blue color intensity provided by b* CIE L*a*b* parameter in agreement with cobalt concentration (**c**).

The presence of CoAl_2_O_4_ was confirmed in our work by analysis of reflectance spectra in the UV-Vis range. Kubelka–Munk functions for compositions I–IV are presented in Figure 6 for dried and pressed tablets of initial composition (a) and for sintered at 1400 °C samples (b). It is clearly seen from the presented data, that color intensity growth significantly after sintering (note y axis scale bar). We attributed adsorption of pressed tablets (Figure 6a) to the presence of mixture of hydrated CoCl_2_·6H_2_O, which absorb in the range of 410–550 nm with peak at 530 nm [37] and anhydrous CoCl_2_ with broad absorption spectrum peaked at about 685 nm linked with not decomposed CoCl_2_-additive, as the obtained spectra were similar to ones with different humidity reported in [38]. Adsorption triplet in Figure 6b at about 540, 590, and 625 nm were ascribed to the [4A2(F) → 4T1(P)] transition in Co^2+^ in a tetrahedral ligand field. The spin-forbidden transition was observed as a small peak at 477 nm. The presence of these peaks is linked to CoAl_2_O_4_ formation by several researchers [39]. Blue color intensity provided by b* chromaticity coordinate growth significantly with Co concentration (Figure 6c). We have observed characteristic color of this spinel as well. The very weak intensity of CoAl_2_O_4_ peaks in the Kubelka–Munk pattern for composition II confirmed the introduction of Co^2+^ ions in t-ZrO_2_ lattice. We could presume, that the first stage of interaction between Co additive with 3Y-TZP-Al_2_O_3_ was linked with solid solution based on t-ZrO_2_ until saturation and at the second stage the excess of Co ions interacted with Al_2_O_3_ with the formation of CoAl_2_O_4_.

### 3.4. The Microstructure Investigations

**Figure 7:**
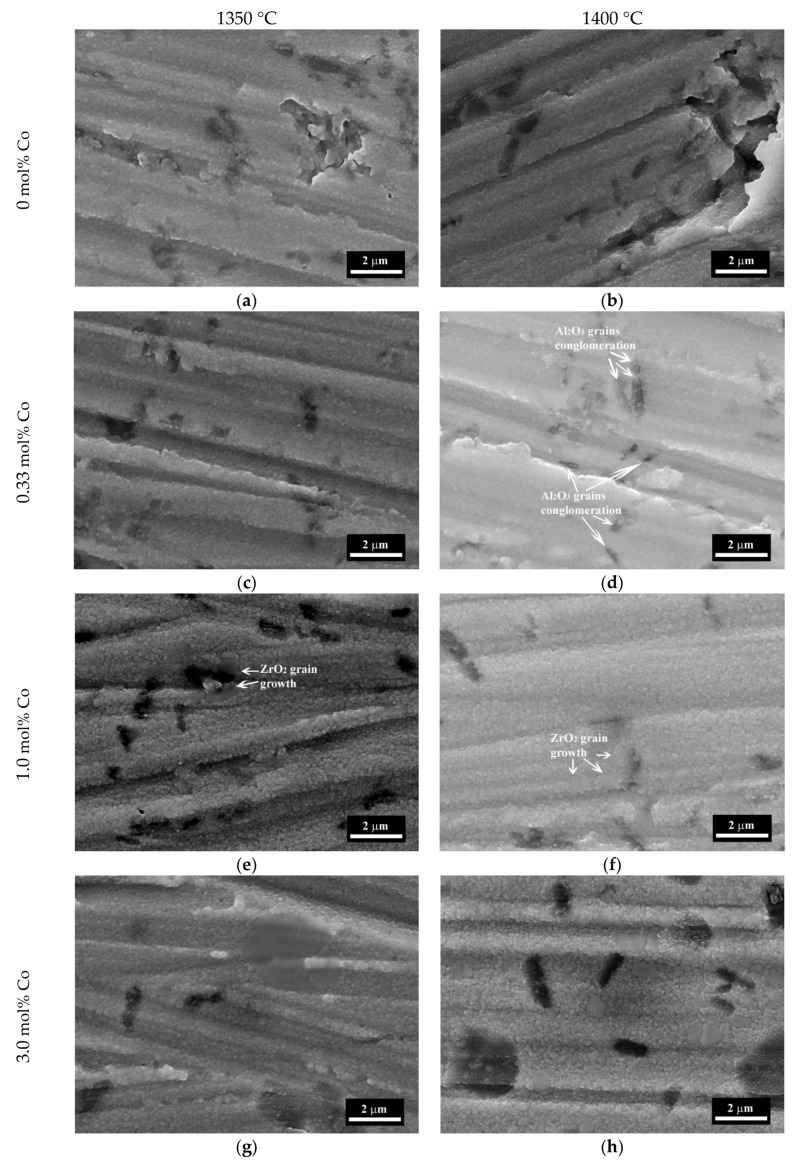
SEM images (SE mode) of 3Y-TZP-Al_2_O_3_ ceramics: composition I (**a**,**b**), composition II (**c**,**d**), composition III (**e**,**f**), composition IV (**g**,**h**) sintered at 1350 (**a**,**c**,**e**,**g**) and 1400 °C (**b**,**d**,**f**,**h**). Dark colors indicate alumina-rich areas; bright colors indicate zirconia-rich areas.

**Figure 8:**
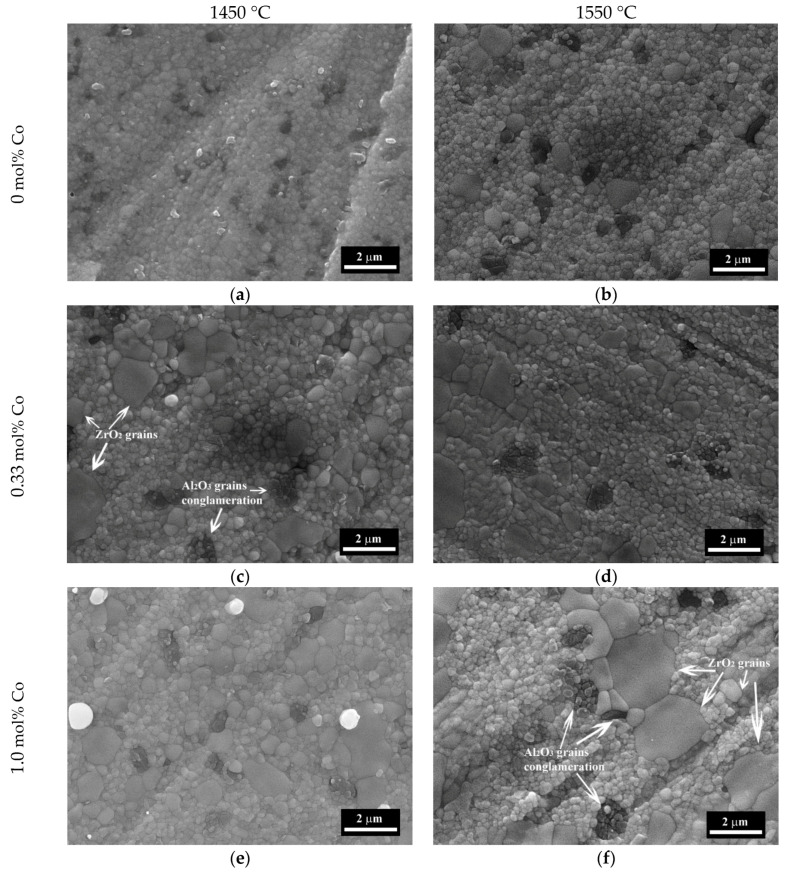

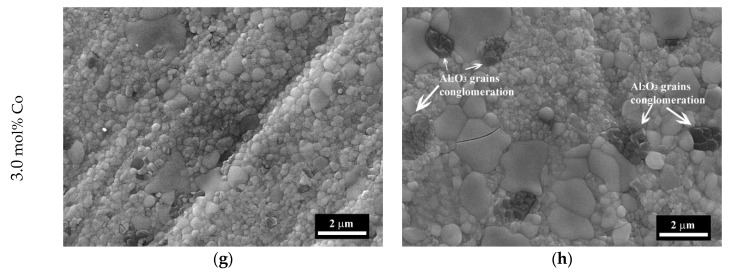
SEM images (SE mode) of 3Y-TZP-Al_2_O_3_ ceramics: composition I (**a**,**b**), composition II (**c**,**d**), composition III (**e**,**f**), composition IV (**g**,**h**) sintered at 1450 (**a**,**c**,**e**,**g**) and 1550 °C (**b**,**d**,**f**,**h**).

The microstructure of the samples I–IV sintered at 1350 and 1400 °C are presented in Figure 7, and for the samples sintered at 1450 and 1550 °C in Figure 8. The compositions I and II sintered at 1350–1400 °C were formed by spherical grains of ZrO_2_ with the average size of 200–400 nm and dark Al_2_O_3_ —enriched particles of the same sizes (Figure 7). The distinction between dark Al_2_O_3_ enriched zones and bright ZrO_2_ zones linked with the differences between the absorptional and reflectional energies of secondary electrons and it was demonstrated previously [40,41]. The increase of the sintering temperature over 1450 °C lead to the appearance of the large grains of ZrO_2_ up to 0.7–0.8 μm for the composition I and 0.8–1.5 μm for composition II. For material without Co additive (composition I) sintered at 1350 °C about 10% porosity was observed with a pore size of 0.1–0.5 μm. Introduction of Co additive resulted in the formation of a dense structure at 1350 °C with open porosity lower than 1% for compositions II, III, and IV. At the same time, we observed the formation of the large grains of ZrO_2_ with a size of 0.5–0.8 μm for composition III with a further increase of size up to 2.0 μm for composition IV at a temperature equal or higher than 1350 °C. Tsukrenko et al. previously had demonstrated that the spinel CoAl_2_O_4_ formation reduced the mutual inhibition of grains growth of Al_2_O_3_ and ZrO_2_ and caused a twofold increase in ZrO_2_ crystallite size for 1200 °C heat-treated ZrO_2_-Y_2_O_3_-CeO_2_-CoO-Al_2_O_3_ powders, obtained by the hydrothermal way [42]. The grains growth promotes the transformation of t-ZrO_2_ into the stable m-ZrO_2_ at low temperature, confirmed by XRD [43].

The increase of the sintering temperature up to 1400–1450 °C for compositions III and IV resulted in further grains growth up to 3.0 μm. The dark grains of Al_2_O_3_ became prism-shaped and formed agglomerates with a size of 3.0–5.0 μm. At 1500 °C there were large grains with the size up to 3.0–5.0 µm and the surface fraction covered by Al-rich grains increased from about 10% to 30% with the increase of Co content. The growth of the sintering temperature resulted in the predomination of the large grains with a size up to 5.0 µm at 1550 °C (Figure 7).

**Figure 9:**
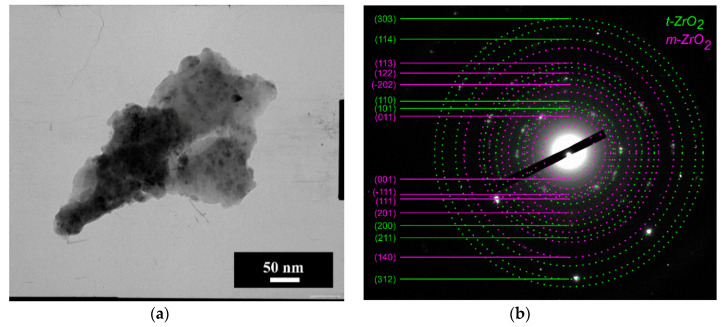

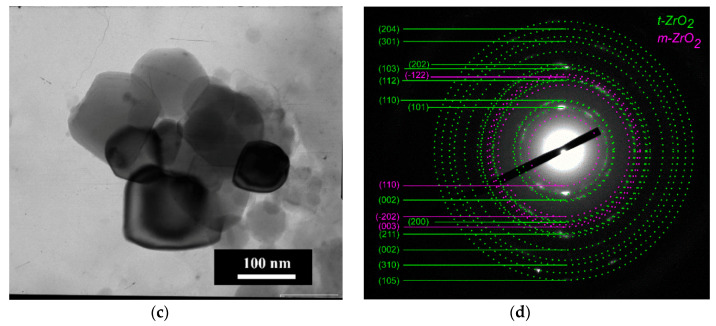
TEM images of 3Y-TZP-Al_2_O_3_ ceramics syntered at 1400 °C: (**a**) composition I; (**c**) composition II and Millers lines of the corresponding ceramics (**b**,**d**).

The use of TEM allows as to investigate the influence of Co on the structure of 3Y-TZP-Al_2_O_3_ ceramics sintered at 1400 °C in more detail (Figure 9). The sample without Co additive was formed by ceramic particles with uniaxial morphology and average grain size of 50 nm. The introduction of 0.33 mol% of Co resulted in the growth of the grains up to 100–150 nm and led to the formation of long and dense contacts between grains. The grains with clear grain boundaries and polyhedral morphology were observed in the TEM micrographs. This indicates the increase of recrystallization with the introduction of cobalt in the ceramic materials. According to electron diffraction data, the ceramic of composition I presented in Figure 9 was formed by both m-ZrO_2_ (with main reflexes assigned to (-111) at 3.16 Å, (111) at 2.84 Å, (001) at 4.96 Å; (011) at 3.62 Å) and t-ZrO_2_ (with main reflexes assigned to (101) at 2.97 Å, (110) at 2.56 Å, (200) at 1.80 Å). The sample of composition II was formed primarily from t-ZrO_2_ with high-intensity lines (101) at 2.97 Å and (110) at 2.55 Å). The intensity of reflexes attributed to m-ZrO_2_ ((-202) at 1.96 Å and (110) at 3.67 Å) was considerably lower. This indicates that the trace amount of the m-ZrO_2_ started to form at 1400 °C.

### 3.5. The Porosity, Density and Mechanical Properties of Ceramic Materials

The results of the samples’ surface investigation by SEM were confirmed by porosity measurement. After sintering at 1350 °C a decrease of open porosity from 9.09% for the composition I to 2.3–2.7% for the compositions II, III, and IV was observed (Table 1). The dense structure with porosity lower than 0.5% was obtained for all samples with Co additive at 1400 °C, at the same time composition I had 4.07%. The composition I was sintered to a dense structure with porosity lower than 1% at 1450 °C. The further increase of sintering temperature did not lead to a further decrease of porosity.

**Figure 10:**
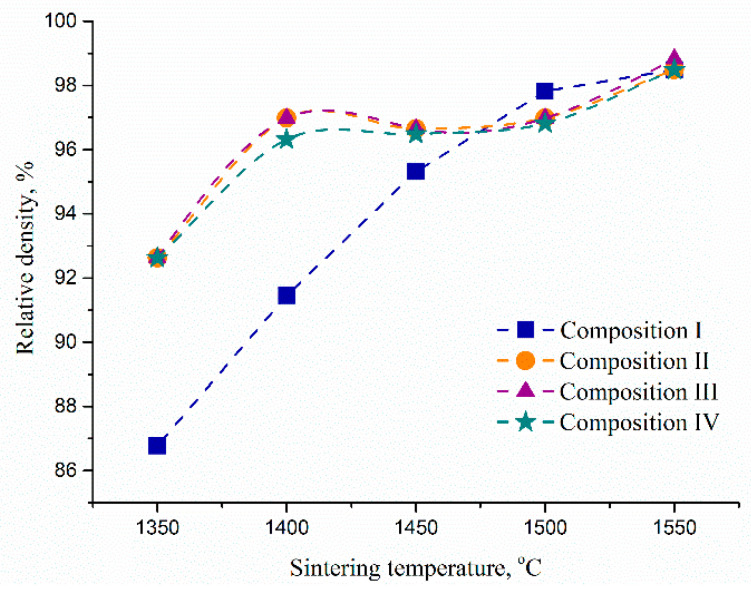
The influence of Co additive on relative density of 3Y-TZP-Al_2_O_3_ sintered at different temperatures.

In order to estimate the total porosity of the samples, the relative density of ceramic bodies was calculated using the additive scheme. The value of a theoretical density was estimated to be 5.97 g/cm^3^, based on the values of 3Y-TZP (6.08 g/cm^3^) [28] and Al_2_O_3_ (3.99 g/cm^3^) [29]. The relative density of the sintered non-porous samples was in the range of 96.0–98.6%, depending on sintering conditions (Figure 10). The samples of composition I reached a high density of over 96% only at 1500 °C. Samples containing Co densified efficiently at T > 1400 °C. A slight unusual decrease in density, observed for the samples II–IV with growing temperature, could be linked to the formation of m-ZrO_2_, provoked by Co. Compositions II–IV were sintered to the almost dense state according to SEM data and open porosity measurements at T > 1400 °C. The difference in open and total porosity for compositions II–IV was not sufficient, this result was in agreement with similar shrinkage behavior of these samples (Figure 3).

**Figure 11:**
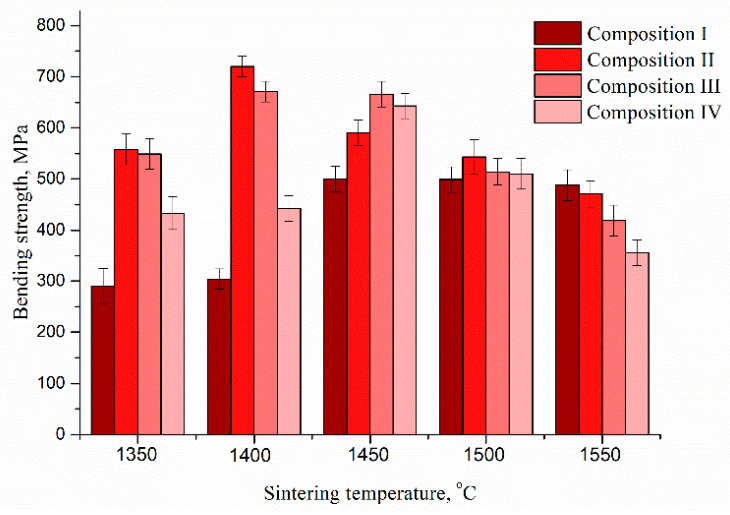
Bending strength of 3Y-TZP-Al_2_O_3_ ceramics depending on sintering temperature and composition.

The results of the bending strength measurement are presented in Figure 11. The introduction of Co resulted in a significant increase in bending strength at sintering temperatures below 1500 °C. Due to dense structure with low porosity and nanoscale grains size, the bending strength at 1350 °C increase twice for composition II compared to the composition I and it is characterized by 590 ± 20 MPa compared to 290 ± 25 MPa for composition I. The increase of Co additive amount resulted in a decline of bending strength due to the formation of large grains and crystallization of the spinel CoAl_2_O_4_ and it was characterized by 480 ± 25 MPa for composition IV. The increase of sintering temperature up to 1400 °C for compositions II and III resulted in the formation of non-porous high strength ceramic materials with bending strength values up to 720 ± 33 MPa and 671 ± 20 MPa respectively, the composition IV was characterized by 471 ± 25 MPa. Further increase of sintering temperature up to 1450 °C resulted in the growth of the bending strength for the composition I up to 500 ± 25 MPa due to a non-porous state. Compositions II, III, and IV were characterized by a noticeable decrease of strength value due to the formation of large grains and the recrystallization of t-ZrO_2_ into m-ZrO_2_. Further growth of sintering temperature resulted in a decline of strength for samples with Co additive due to further recrystallization with the appearance of a coarse-grained structure with a significant amount of m-ZrO_2_. The composition I demonstrated the lowest degree of recrystallization and grains growth after sintering at 1550 °C. As a result, it had a higher bending strength than Co-contained compositions. This indicates that the sintering temperature of Co-contained composite materials should not exceed 1450 °C.

The measurement of microhardness was performed on the samples sintered in the temperature range of 1350–1450 °C, which characterized by predominate formation of t-ZrO_2_. For the composition I the values increase from 6.3 ± 0.1 to 9.2 ± 0.2 GPa with the growth of the sintering temperature from 1350 to 1450 °C linked with the corresponding decrease of the ceramic samples porosity. The cobalt addition resulted in a significant increase of the microhardness up to 8.3 ± 0.2 GPa for 1350 °C and 10.3 ± 0.2 GPa for 1450 °C for composition II. The increase of Co additive amount resulted in the considerable increase of the microhardness up to 11.6 ± 0.2 GPa for composition III at 1450 °C linked with the formation of spinel CoAl_2_O_4_ grains according to SEM, which characterized by higher microhardness comparing to t-ZrO_2_ (Figure 4) [44]. Composition IV was characterized by 10.6 ± 0.2 GPa at 1450 °C due to the simultaneous formation of CoAl_2_O_4_ and a coarse-grained structure based on the high amount of the m-ZrO_2_ [45].

### 3.6. The Results of In Vitro Investigations

The results of in vitro investigations demonstrated, that the samples based on the composite ceramic materials of 3Y-TZP-Al_2_O_3_ are non-toxic: after 24 h of incubation of human sarcoma cells with the extracts of these samples, the size of PVC was close to the control values—amounting to 89–102.9%, and the toxicity index was 1.2–11.0%, respectively. The pH values of the extracts demonstrated a neutral reaction. With an increase in the cell growth time to 48 and 72 h the identified trend continued: the MG-63 population grew uniformly without obvious signs of toxicity (lower, than 30%) in the presence of extracts of the all studied materials as evidenced by the size of the pool of viable cells and toxicity index (Table 2).

**Figure 12:**
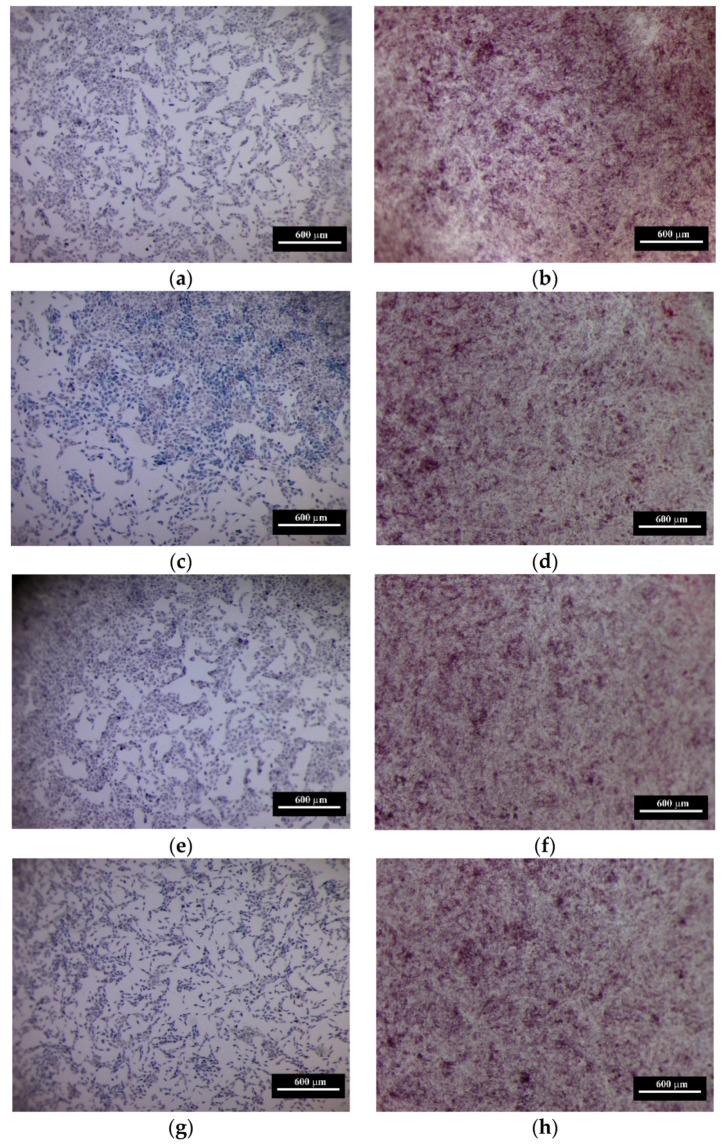

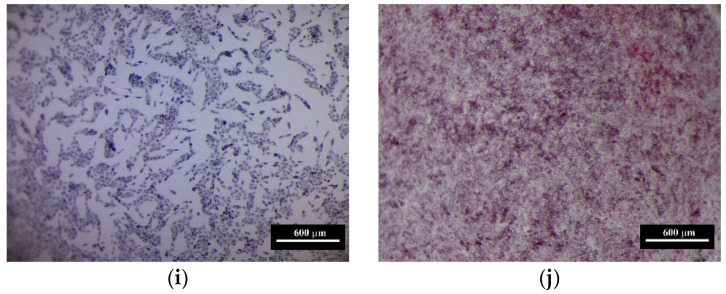
The cell population of human sarcoma MG-63 after 24 (**a**,**c**,**d**,**e**,**g**,**i**) and 72 (**b**,**d**,**f**,**h**,**j**) hours of CGM (control, **a**,**b**) and in the presence of extracts of composite ceramic samples with 0.0 (**c**,**d**), 0.33 (**e**,**f**), 1.0 (**h**,**g**) and 3.0 (**i**,**j**) mol% Co, respectively.

Cell populations of human sarcoma after 24 and 72 h are presented in Figure 12. This result demonstrates that the introduction of Co did not lead to cytotoxicity and demonstrated cytocompatibility. Previously Santos et al. [46] have shown, that 3Y-TZP reinforced by Al_2_O_3_ particles sintered at 1600 °C was promising for bioceramics application. The influence of Co doping on the biocompatibility of Y-TZP or Y-TZP-Al_2_O_3_ composite materials was not discussed up to day. The Co-Cr alloys are widely used for orthopedic implants [47]. The investigation of the influence of Co^2+^ ions, which could be released from metal implants, on the MG-63 cells demonstrated the concentration and time dependent cytotoxic effect [48]. It should be underlined, that CoAl_2_O_4_ demonstrated the lowest Co bioaccessibility compared to other Co-containing compounds—Co oxides and salts, in all fluid equivalent, including Gastric (pH 1.5), Alveolar (pH 7.4), Serum, Lysosomal (pH 4.5) [47,49]. CoAl_2_O_4_ was considered as a promising alternative to Co_2_O_3_ nanoparticles pigment due to its safety [50]. At the same time recently Co oxides-containing bioglasses had demonstrated the improvement of angiogenesis [51] and osteogenesis [52]. Additionally, in in vitro tests, Co-contained bioglasses demonstrated no cytotoxicity up to 72 h, and cobalt incorporation in the bioactive glass did not affect the mitochondrial activity of human umbilical vein endothelial cells (HUVECs) [51]. In [53] 2 and 5 mol% Co-substituted β-tricalcium phosphate ceramics (β-TCP) demonstrated cytocompatibility of human bone marrow mesenchymal stem cells and HUVECs, and showed an improved in vitro angiogenic potential as compared with pure β-TCP. Thus, the introduction of Co in the form of dopant in the bioceramic and bioglass did not cause cytotoxicity and resulted in the improvement of bone repair. The analysis of OD and PVC in our work demonstrated that the increase of cobalt content resulted in the slight growth of the cellular viability and this trend became more visible at 72 h (Figure 11). In all cases, the introduction of Co additive resulted in more extensive cell growth compared to pure 3Y-TZP-Al_2_O_3_. Our data demonstrated that Co-doped 3Y-TZP-Al_2_O_3_ ceramics developed in this work are cytocompatible and could be considered as a potential ceramic material for biomedical appliction [54].

### 3.7. The DLP Printed and Sintered Samples

**Figure 13:**
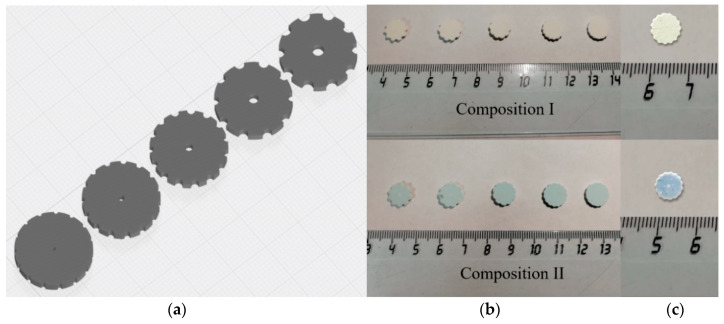
The CAST model (**a**), DLP printed (**b**) and sintered (**c**) optical photo samples.

**Figure 14:**
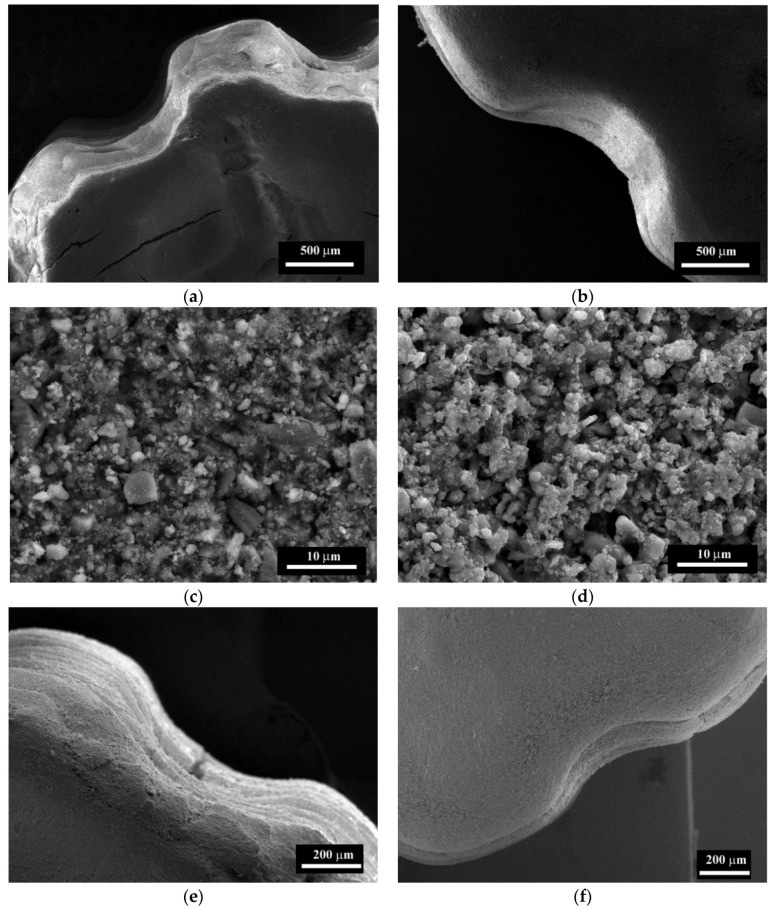

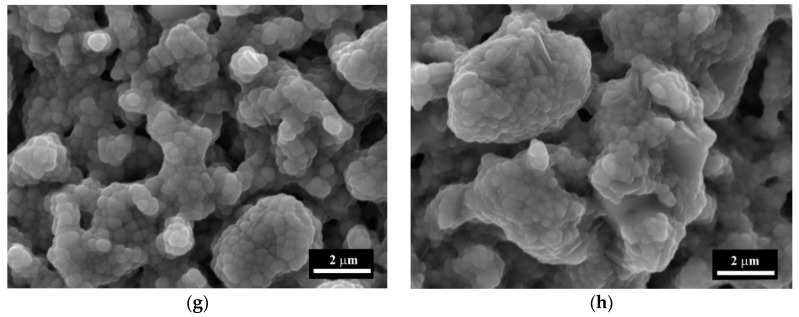
SEM images of composition I (**a**,**c**,**e**,**g**) and composition II (**b**,**d**,**f**,**h**) after printing **(a**,**b**,**c**,**d**) and sintering at 1450 °C (**e**,**f**,**g**,**h**).

The optical photo of the printed samples and sintered ceramics, as well as computer models, are presented in Figure 13. Presented shapes of the printed models were selected to investigate ceramics composition influence on printing quality. Addition of Co allows to increase printing resolution, the printed samples of 3Y-TZP-Al_2_O_3_ without Co (composition I) are characterized by pronounced layering and flowing of the green bodies. It could be linked with the low viscosity of the slurry and not sufficient adhesion of the layers. The holes of the green body were significantly reduced due to light scattering and extra polymerization of the slurry. The sizes of the holes were lower than the adjusted by the CAST model for approximately 30% and gradually disappeared when the diameter of the expected ones was smaller than 200 µm. The samples with 0.33 mol% of Co additive are characterized by the smooth border and the decreased amount of the defects. It should be linked with the improved adhesion between layers of the composition II-based slurry. At the same time, according to Borlaf et.al. [26] the good adhesion is linked with sufficient printing layer thickness and indicated the optimal resolution of the printing. The microstructure of as-printed green bodies was dense and formed by homogeneously distributed ceramic particles in the polymer matrix (Figure 14a–d).

After the sintering at 1450 °C the linear shrinkage for the composition I was in the range 23–25%, for composition II it was in the range 25–27%. According to SEM, the sintered samples were porous, due to the removing of a high amount of photopolymer during the debinding processes and low volume fraction of the ceramics, but in the case of composition II, the growth of the density was observed. The quality of the surface of the sintered samples was improved with Co introduction (Figure 14e,f). The specimens with 0.33 mol% of Co characterized by flowing twists and clean parts, compared to the rough layered structure of composition I. The grains sizes ranged between 200–400 nm and the incorporation of large grains with a size of 2 µm (Figure 14g,h). Similar effects were observed for pre-pressed samples of Composition II shown in Figure 8.

## 4. Discussion

In our work, we demonstrated the influence of the Co oxide amount on the sintering intensification, phase formation, mechanical and biological properties, as well as efficiency as a ceramic filler for 3D manufacturing via DLP technologies. The solid state sintering of the t-ZrO_2_ materials with low Al_2_O_3_ content (5%) is a challenge up to date. Ye et al. [34] demonstrated the decrease of the relative density with an increase of zirconia content up to 85% in the yttria-stabilized ZrO_2_/Al_2_O_3_ composition materials and they could not achieve the full density for the 5 mol% Al_2_O_3_-3Y-TZP composite ceramics after the sintering during 15 h at 1450 °C. For example, sintering at 1600 °C was required for the production of 3Y-TZP bioceramic material reinforced by Al_2_O_3_ particles. The high temperature and long sintering limit the application of this type of ceramic materials. Among the oxide additives, Co oxide is a promising sintering promoter and activator for interaction with visible irradiation during the 3D manufacturing [9]. The Co oxides were already demonstrated as an effective sintering additive for pure cubic ZrO_2_, but the influence of Co on the solid state sintering of composite materials based on t-ZrO_2_ and Al_2_O_3_ was not investigated.

The interaction between Co and ZrO_2_-Al_2_O_3_ ceramic is a complex process. It is known [21,22], that Y-stabilized cubic ZrO_2_ formed solid solution with Co oxides, moreover two types of substitution sites were found. This led to a decrease of cell volume at low concentration of Co (filling of the substitution positions) and a further increase in lattice parameter due to consequent occupation of the interstitial sites. This process is known to intensify the solid state sintering. We managed to observe for the first time the same process of the solid solution formation for t-ZrO_2_ during the sintering (Figure 5). At the same time, Co oxide can react with Al_2_O_3_ forming CoAl_2_O_4_ spinel at high temperature [34]. Competition between these two processes led to the complex distribution of Co depending on a lot of parameters of ceramic production processes, for example, amount of Al_2_O_3_, sintering temperature, powders surface area, and others. One important point is the potential toxicity of Co oxides, and complete conversion of Co into solid solution and spinel led to the production of biocompatible ceramic material, as it was confirmed by our in vitro investigation.

In our work, we observed the increase of the linear shrinkage and grains growth with the introduction of Co oxide additive. The introduction of a low amount of Co resulted in significant sintering intensification with the preservation of nanoscale grains size. We linked the effect of low Co amount on the activated sintering with the formation of substitution type solid solutions of Co in 3Y-TZP.

The further increase of Co concentration resulted in the formation of both substitutional and interstitial sites solid solution. At the same time, the formation of CoAl_2_O_4_ when 3 mol% of Co was introduced resulted in the most significant grain growth at the low temperatures. Previously the decrease of ceramic density was demonstrated with the grain growth in the case of liquid-phase sintering Y-TZP with SiO_2_ [32]. As the amount of Co increased, the growth of the grains occurred more noticeable at low temperature. This was confirmed by SEM data: the grains with size up to 1 μm at T = 1350 °C were observed in the compositions III and IV, and the amount of these large grains increased with the growth of Co concentration. Additionally, we observed the destabilization of the phase composition resulted in the increase of m-ZrO_2_ amount with Co content growth. This effect is probably linked with the formation of large grains [43].

The tendency of ZrO_2_ grain growth linked with the introduction of Co was demonstrated for all additives amounts at high temperatures. At the same time, when the lowest amount of Co-0.33 mol% (composition II) was introduced, the grains remained nanoscale at 1350–1400 °C and became microscale at 1450 °C, compared to observed for composition IV after sintering at 1350 °C grains of the 1–2 µm. Samples prepared from composition II demonstrated the highest bending strength after the sintering at 1400 °C–720 ± 33 MPa. It was provided due to the formation of the dense microstructure consisted of predominately nanosized grains of t-ZrO_2_. Further growth of the sintering temperature resulted in an increase of the m-ZrO_2_ amount, grain size, and led to a recession of the mechanical properties. In addition, we have demonstrated the possibility of the CoAl_2_O_4_ formation to increase the microhardness up to 11.06 GPa at 1450 °C.

Ceramic samples based on the ZrO_2_-Al_2_O_3_ system were successfully obtained via 3D printing DLP based stereolithography for the first time. The data on materials extrusion based 3D production of ZrO_2_–20% Al_2_O_3_ ceramics was reported previously, but the resolution of this technique is limited and an 800 μm thickness layer was obtained [55]. In our work, the printing depth was 35 µm per layer thanks to DLP technology. The first data of DLP ZrO_2_-Al_2_O_3_ species production via commercial 3D printer demonstrated the improvement of the resolution and border quality of as-printed and sintered species via Co additive introduction, as it was demonstrated earlier for pure 3Y-TZP without Al_2_O_3_ [9]. The reached results are promising for the development of 3D printing bioceramics technology.

## 5. Conclusions

In the present paper, the Y-TZP-5Al_2_O_3_ nanopowders were synthesized via a precipitation method. The introduction of Co oxide resulted in a significant intensification of the sintering processes due to the formation of Y-TZP-based solid solution and CoAl_2_O_4_ spinel. In the presence of 0.33 mol% of Co tetragonal structure of ZrO_2_ was preserved up to 1400 °C. The non-porous dense structure was formed with bending strength up to 720 MPa. The introduction of 1.0–3.0 mol% of Co resulted in the formation of CoAl_2_O_4_ spinel with the improvement of ceramic microhardness up to 11.06 GPa after the sintering at 1450 °C. Further increase of sintering temperature led to noticeable grains growth, resulting in a decrease in mechanical properties. According to an in vitro test (ISO 10993.5-99), ceramic materials with Co oxide additive did not demonstrate cytotoxicity and are promising for bioceramic materials development.

The slurry based on the oligoester acrylate and synthesized powders were obtained and applied for complex shape samples 3D manufacturing via DLP based stereolithographic technology. The introduction of 0.33 mol% of Co resulted in the quality improvement of both green body and sintered samples. The reached results are promising for the development of high-resolution 3D manufacturing technology of Y-TZP-Al_2_O_3_ bioceramic materials.

## Figures and Tables

**Table 1:** The open porosity values (%) of ceramics samples depending on the sintering temperature.

Materials	Sintering Temperature, °C				
	1350	1400	1450	1500	1550
Composition I	9.09	4.07	0.06	0.12	0
Composition II	2.40	0.06	0.12	0.08	0
Composition III	2.74	0.06	0.22	0.08	0
Composition IV	2.27	0.24	0.16	0.09	0.05

**Table 2:** The results of in vitro investigations: the pH of the extracts, the values of the optical density (OD) of the formazan solution (MTT test), the pool of viable cells (PVC) and the toxicity index (TI) during the cultivation of human sarcoma cells of the MG-63 cell line in the presence of the extracts of samples based on the composite ceramic materials of 3Y-TZP-Al_2_O_3_.

Materials	pH Value of Extract, CGM	Time (h)								
		24			48			72		
		OD, a.u. (M ± m)	PVC, %	TI, %	OD, a.u. (M ± m)	PVC, %	TI, %	OD, a.u. (M ± m)	PVC, %	TI, %
Composition I	7.4	0.219 ± 0.002	89.4	10.6	0.440 ± 0.010	90.9	9.1	0.633 ± 0.016	80.8	19.2
Composition II	7.4	0.242 ± 0.002	98.8	1.2	0.446 ± 0.001	92.1	7.9	0.632 ± 0.005	80.7	19.3
Composition III	7.4	0.252 ± 0.04	102.9	0.0	0.454 ± 0.006	93.8	6.2	0.651 ± 0.006	83.1	16.9
Composition IV	7.4	0.241 ± 0.007	98.4	1.6	0.457 ± 0.003	94.4	5.6	0.678 ± 0.002	86.6	13.4
Control (CGM)	7.3	0.245 ± 0.009	100.0	0.0	0.484 ± 0.003	100.0	0.0	0.783 ± 0.007	100.0	0.0

## References

[1] Manicone P., Rossi Iommetti P., Raffaelli L. (2007). An overview of zirconia ceramics: Basic properties and clinical applications. J. Dent..

[2] Afzal A. (2014). Implantable zirconia bioceramics for bone repair and replacement: A chronological review. Mater. Express.

[3] Kagawa M., Kikuchi M., Syono Y., Nagae T. (1983). Stability of Ultrafine Tetragonal ZrO_2_ Coprecipitated with Al_2_O_3_ by the Spray-ICP Technique. J. Am. Ceram. Soc..

[4] Wu Z.K., Li N., Jian C., Zhao W.Q., Yan J.Z. (2013). Low temperature degradation of Al_2_O_3_-doped 3Y-TZP sintered at various temperatures. Ceram. Int..

[5] Chevalier J. (2006). What future for zirconia as a biomaterial?. Biomaterials.

[6] Daguano J., Santos C., Souza R., Balestra R., Strecker K., Elias C. (2007). Properties of ZrO_2_–Al_2_O_3_ composite as a function of isothermal holding time. Int. J. Refract. Met. Hard Mater..

[7] Abden M., Afroze J., Qadir M., Gafur M., Chowdhury F. (2015). Correlation among composition, microstructure and mechanical properties of ZrO_2_ (Y_2_O_3_)/Al_2_O_3_ composite ceramics. Int. J. Mater. Eng. Innov..

[8] Hwang K., Zhao J., Kim J., Lee J. (2015). Dispersion of Nano Size ZrO2 in Al_2_O_3_/ZrO_2_ Ceramics by Hydrolysis. Procedia Manuf..

[9] Li Y., Wang M., Wu H., He F., Chen Y., Wu S. (2019). Cure behavior of colorful ZrO_2_ suspensions during Digital light processing (DLP) based stereolithography process. J. Eur. Ceram. Soc..

[10] Hong J., Gao L., Torre S., Miyamoto H., Miyamoto K. (2000). Spark plasma sintering and mechanical properties of ZrO_2_(Y_2_O_3_)–Al_2_O_3_ composites. Mater. Lett..

[11] Smirnov V., Krylov A., Smirnov S., Goldberg M., Antonova O., Shvorneva L., Barinov S. (2017). Study of liquid-phase sintering of materials based on zirconium dioxide containing alumina. Inorg. Mater. Appl. Res..

[12] Oelgardt C., Anderson J., Heinrich J., Messing G. (2010). Sintering, microstructure and mechanical properties of Al_2_O_3_–Y_2_O_3_–ZrO_2_ (AYZ) eutectic composition ceramic microcomposites. J. Eur. Ceram. Soc..

[13] Gil-Flores L., Salvador M., Penaranda-Foix F., Fernández A., Suarez M., Rosa R., Veronesi P., Leonelli C., Borrell A. (2019). Microstructure and mechanical properties of 5.8 GHz microwave-sintered ZrO_2_/Al_2_O_3_ ceramics. Ceram. Int..

[14] Flegler A., Burye T., Yang Q., Nicholas J. (2014). Cubic yttria stabilized zirconia sintering additive impacts: A comparative study. Ceram. Int..

[15] Obolkina T., Goldberg M., Smirnov V., Smirnov S., Titov D., Konovalov A., Kudryavtsev E., Antonova O., Barinov S., Komlev V. (2020). Increasing the Sintering Rate and Strength of ZrO_2_–Al_2_O_3_ Ceramic Materials by Iron Oxide Additions. Inorg. Mater..

[16] He R., Liu W., Wu Z., An D., Huang M., Wu H., Jiang Q., Ji X., Wu S., Xie Z. (2018). Fabrication of complex-shaped zirconia ceramic parts via a DLP- stereolithography-based 3D printing method. Ceram. Int..

[17] Zhang K., He R., Ding G., Feng C., Song W., Fang D. (2020). Digital light processing of 3Y-TZP strengthened ZrO_2_ ceramics. Mater. Sci. Eng. A.

[18] Osman R., van der Veen A., Huiberts D., Wismeijer D., Alharbi N. (2017). 3D-printing zirconia implants; a dream or a reality? An in-vitro study evaluating the dimensional accuracy, surface topography and mechanical properties of printed zirconia implant and discs. J. Mech. Behav. Biomed. Mater..

[19] Ding G., He R., Zhang K., Xie C., Wang M., Yang Y., Fang D. (2019). Stereolithography-based additive manufacturing of gray-colored SiC ceramic green body. J. Am. Ceram. Soc..

[20] Llusar M., Forés A., Badenes J., Calbo J., Tena M., Monrós G. (2001). Colour analysis of some cobalt-based blue pigments. J. Eur. Ceram. Soc..

[21] Hartmanová M., Hanic F., Tunega D. (1998). Structural and electro-optical properties of Co-doped yttria-stabilized zirconia. Chem. Pap..

[22] Lewis G., Atkinson A., Steele B. (2001). Journal search results—Cite This for Me. J. Mater. Sci. Lett..

[23] Czarnek K., Terpiłowska S., Siwicki A. (2015). Review paper Selected aspects of the action of cobalt ions in the human body. Cent. Eur. J. Immunol..

[24] Aherwar A., Singh A.K., Patnaik A. (2016). Cobalt Based Alloy: A Better Choice Biomaterial for Hip Implants. Trends Biomater. Artif. Organs.

[25] Rittidech A., Somrit R., Tunkasiri T. (2013). Effect of adding Y_2_O_3_ on structural and mechanical properties of Al_2_O_3_–ZrO_2_ ceramics. Ceram. Int..

[26] Borlaf M., Serra-Capdevila A., Colominas C., Graule T. (2019). Development of UV-curable ZrO_2_ slurries for additive manufacturing (LCM-DLP) technology. J. Eur. Ceram. Soc..

[27] de l’Eclairage, Commision Internationale (1978). Recommendations on Uniform Color Spaces, Color-Difference Equations, Psychometric Color Terms.

[28] Borrell A., Salvador M.D., Rayón E., Penaranda-Foix F.L. (2012). Improvement of microstructural properties of 3Y-TZP materials by conventional and non-conventional sintering techniques. Ceram. Int..

[29] Sumita S. (1991). Influence of oxide additives, firing temperature, and dispersing media on sintered Al_2_O_3_. J. Ceram. Soc. Jpn..

[30] Maca K., Trunec M., Chmelik R. (2007). Processing and properties of fine-grained transparent MgAl_2_O_4_ ceramics. Ceram. Silik..

[31] Viswanath B., Ravishankar N., Nayar S., Sinha A. (2004). Synthesis, Sintering and Microstructural Characterization of Nanocrystalline Hydroxyapatite Composites. MRS Proc..

[32] Mosmann T. (1983). Rapid colorimetric assay for cellular growth and survival: Application to proliferation and cytotoxicity assays. J. Immunol. Methods.

[33] Lakusta M., Danilenko I., Konstantinova T., Volkova G., Nosolev I., Gorban O., Syniakina S., Burkhovetskiy V. (2017). The Effect of a Small Amount SiO_2_ on Sintering Kinetics of Tetragonal Zirconia Nanopowders. Nanoscale Res. Lett..

[34] Ye Y., Li J., Zhou H., Chen J. (2008). Microstructure and mechanical properties of yttria-stabilized ZrO_2_/Al_2_O_3_ nanocomposite ceramics. Ceram. Int..

[35] Chiou Y., Lin S. (1996). Influence of CoO and Al_2_O_3_ on the phase partitioning of ZrO_2_-3 mol% Y_2_O3. Ceram. Int..

[36] Cava S., Tebcherani S.M., Pianaro S.A., Paskocimas C.A., Longo E., Varela J.A. (2006). Structural and spectroscopic analysis of -Al_2_O_2_ to α-Al2O3-CoAl_2_O_4_ phase transition. Mater. Chem. Phys..

[37] Tsigara A., Mountrichas G., Gatsouli K., Nichelatti A., Pispas S., Madamopoulos N., Vainos N.A., Du H.L., Roubani-Kalantzopoulou F. (2007). Hybrid polymer/cobalt chloride humidity sensors based on optical diffraction. Sens. Actuators B Chem..

[38] You M.H., Yan X., Zhang J., Wang X.X., He X.X., Yu M., Ning X., Long Y.Z. (2017). Colorimetric humidity sensors based on electrospun polyamide/CoCl_2_ nanofibrous membranes. Nanoscale Res. Lett..

[39] Zheng W., Zou J. (2015). Synthesis and characterization of blue TiO_2_/CoAl_2_O_4_ complex pigments with good colour and enhanced near-infrared reflectance properties. RSC Adv..

[40] Kim D.J., Lee M.H., Lee D.Y., Han J.S. (2000). Mechanical properties, phase stability, and biocompatibility of (Y, Nb)-TZP/Al_2_O_3_ composite abutments for dental implant. J. Biomed. Mater. Res. Off. J. Soc. Biomater. Jpn. Soc. Biomater. Aust. Soc. Biomater. Korean Soc. Biomater..

[41] Podzorova L.I., Shvorneva L.I., Il’icheva A.A., Alad’ev N.A., Pen’kova O.I. (2013). Microstructure and phase composition of ZrO_2_-CeO_2_-Al_2_O_3_ materials modified with MgO and Y_2_O_3_. Inorg. Mater..

[42] Tsukrenko V., Dudnik E., Shevchenko A. (2012). Nanocrystalline zirconia based powders synthesized by hydrothermal method. Process. Appl. Ceram..

[43] Chevalier J., Gremillard L., Virkar A., Clarke D. (2009). The Tetragonal-Monoclinic Transformation in Zirconia: Lessons Learned and Future Trends. J. Am. Ceram. Soc..

[44] Pavia A., Laurent C., Weibel A., Peigney A., Chevallier G., Estournès C. (2012). Hardness and friction behavior of bulk CoAl_2_O_4_ and Co–Al_2_O_3_ composite layers formed during Spark Plasma Sintering of CoAl_2_O_4_ powders. Ceram. Int..

[45] Trunec M. (2008). Effect of grain size on mechanical properties of 3Y-TZP ceramics. Ceramics–Silikáty.

[46] Santos C., Teixeira L.H.P., Daguano J.K.M.F., Rogero S.O., Strecker K., Elias C.N. (2009). Mechanical properties and cytotoxicity of 3Y-TZP bioceramics reinforced with Al_2_O_3_ particles. Ceram. Int..

[47] Lison D., Van den Brule S., Van Maele-Fabry G. (2018). Cobalt and its compounds: Update on genotoxic and carcinogenic activities. Crit. Rev. Toxicol..

[48] Fleury C., Petit A., Mwale F., Antoniou J., Zukor D.J., Tabrizian M., Huk O.L. (2006). Effect of cobalt and chromium ions on human MG-63 osteoblasts in vitro: Morphology, cytotoxicity, and oxidative stress. Biomaterial.

[49] Stopford W., Turner J., Cappellini D., Brock T. (2003). Bioaccessibility testing of cobalt compounds. J. Environ. Monit..

[50] Álvarez-Docio C.M., Reinosa J.J., Del Campo A., Fernández J.F. (2017). 2D particles forming a nanostructured shell: A step forward cool NIR reflectivity for CoAl_2_O_4_ pigments. Dye. Pigment..

[51] Barrioni B.R., de Laia A.G.S., Valverde T.M., da Mata Martins T.M., Caliari M.V., de Sa M.A., de Goes A.M., de Magalhães Pereira M. (2018). Evaluation of in vitro and in vivo biocompatibility and structure of cobalt-releasing sol-gel bioactive glass. Ceram. Int..

[52] Quinlan E., Partap S., Azevedo M.M., Jell G., Stevens M.M., O’Brien F.J. (2015). Hypoxia-mimicking bioactive glass/collagen glycosaminoglycan composite scaffolds to enhance angiogenesis and bone repair. Biomaterials.

[53] Zhang M., Wu C., Li H., Yuen J., Chang J., Xiao Y. (2012). Preparation, characterization and in vitro angiogenic capacity of cobalt substituted β-tricalcium phosphate ceramics. J. Mater. Chem..

[54] Ciapetti G., Cenni E., Pratelli L., Pizzoferrato A. (1993). In vitro evaluation of cell/biomaterial interaction by MTT assay. Biomaterials.

[55] Promakhov V., Zhukov A., Dubkova Y., Zhukov I., Kovalchuk S., Zhukova T., Olisov A., Klimenko V., Savkina N. (2018). Structure and properties of ZrO_2_–20% Al_2_O_3_ ceramic composites obtained using additive technologies. Materials.

